# Clinical Manifestations and Diagnosis of Acromegaly

**DOI:** 10.1155/2012/540398

**Published:** 2012-02-01

**Authors:** Gloria Lugo, Lara Pena, Fernando Cordido

**Affiliations:** ^1^Department of Endocrinology, University Hospital A Coruña, Xubias deArriba 84, 15006 A Coruña, Spain; ^2^Department of Investigation, University Hospital A Coruña, Xubias de Arriba 84, 15006 A Coruña, Spain; ^3^Department of Medicine, University of A Coruña, 15006 A Coruña, Spain

## Abstract

Acromegaly and gigantism are due to excess GH production, usually as a result of a pituitary adenoma. The incidence of acromegaly is 5 cases per million per year and the prevalence is 60 cases per million. Clinical manifestations in each patient depend on the levels of GH and IGF-I, age, tumor size, and the delay in diagnosis. Manifestations of acromegaly are varied and include acral and soft tissue overgrowth, joint pain, diabetes mellitus, hypertension, and heart and respiratory failure. Acromegaly is a disabling disease that is associated with increased morbidity and reduced life expectancy. The diagnosis is based primarily on clinical features and confirmed by measuring GH levels after oral glucose loading and the estimation of IGF-I. It has been suggested that the rate of mortality in patients with acromegaly is correlated with the degree of control of GH. Adequately treated, the relative mortality risk can be markedly reduced towards normal.

## 1. Introduction

Acromegaly and gigantism are due to excess GH production, usually caused by a pituitary adenoma. The diagnosis is often preceded by around 10 years of active but unrecognized disease [1]. Pituitary adenomas account for approximately 15% of all intracranial tumors. The incidence of acromegaly is approximately 5 cases per million per year and the prevalence is 60 cases per million [2]. Somatotroph adenomas are monoclonal in origin and develop from genetic changes. Other contributors that could facilitate the expansion of the somatotroph tumor cells are hypothalamic GHRH or somatostatin, paracrine growth factors, and ghrelin [1, 3]. Recent data suggest the importance of the role of ghrelin in the pathophysiological regulation of GH [4–6]. Over 90% of patients with acromegaly have a benign monoclonal pituitary adenoma, which are not surrounded by hyperplasic tissue. Densely granulated adenomas grow slowly and occur in patients over the age of 50. Sparsely granulated adenomas grow faster and occur in younger patients.

 Some 25% of GH-secreting adenomas also secrete prolactin. These include dimorphic adenomas with GH and prolactin cells, monomorphic mammosomatotroph adenomas, and more primitive acidophilic cell adenomas. Multicellular or unicellular immunoreactivity is common, especially for the alpha subunit of glycoprotein hormones. The secretion of other hormones has very uncommon clinical consequences [7]. Over 70% of somatotroph adenomas are macroadenomas at diagnosis, but somatotropic cell carcinoma is exceptional. The ectopic secretion of GH is equally outstanding. Familial syndromes of acromegaly are rare. Excessive production of GHRH from central hypothalamic sources (usually gangliocytomas) or peripheral sources can lead to somatotroph hyperplasia and acromegaly [8].

The manifestations of acromegaly are varied, including acral and soft tissue overgrowth, joint pain, diabetes mellitus, hypertension, and heart and respiratory failure. It has been suggested that the mortality rate in acromegalic patients is correlated with the degree to which GH hypersecretion is controlled. The decreased survival rate associated with acromegaly can be normalized by successful surgical and adjunctive therapy. The mortality rate of cancer can be stratified according to the level of GH treatment and may be similar to that of the general population. Analyzing mortality determinants, around 60% of acromegalic patients die from cardiovascular disease, 25% from respiratory causes, and 15% from neoplasias [9].

## 2. Clinical Manifestations

Clinical manifestations in each patient depend on the levels of GH and IGF-I, age, tumor size, and the delay in diagnosis. The typical features of acromegaly slowly develop over years; around 40% of acromegalic patients are diagnosed by internists, ophthalmologists if they have visual disturbances, dentists due to maxillary teeth separation, mandibular prognathism, and overbite, gynecologists due to menstrual irregularities and infertility, rheumatologists if they suffer form joint problems, or pulmonologist if they have obstructive sleep apnea [10] (Table 1). GH is also implicated in other actions that are not mediated by IGF-I, such as anti-insulin, lipolytic, and antinatriuretic effects. A recent study found that excess GH in humans is associated with increased activity of the epithelial sodium channel, and this could contribute to the volume expansion and soft tissue manifestations seen in acromegaly [11]. GH secreting pituitary adenomas in children before growth is complete cause gigantism. Pituitary gigantism is very rare: in a total sample of 2367 children and adolescents with pituitary adenomas, only 0.6% had gigantism [12].

The growth of the pituitary adenoma may compress local structures and cause neurological symptomatology and visual disturbances. Somatotroph adenomas grow slowly, and patients presenting these adenomas are usually older than 50 years. Changes in appearance derive from skeletal growth and soft tissue enlargement, which is subtle in the early stage of the disease. Visceromegalies are common, in the form of goiter, hepatomegaly, splenomegaly, and macroglossia. Facial changes include large lips and noses, frontal skull bossing and cranial ridges, mandibular overgrowth with prognathism, maxillary widening with teeth separation, jaw malocclusion, and overbite. Growth occurs on acral parts, with an increase in shoe and ring size [13].

The clinical manifestations of acromegaly include skin changes such as hyperhidrosis, oily skin, and unpleasant odor, which are due to the deposit of glycosaminoglycans. Pigmented skin tags over the trunk are common in patients with acromegaly; it is not clear whether GH/IGF-I excess causes skin tags directly, or whether they arise as a consequence of insulin resistance and dyslipidemia. Acanthosis nigricans develop in patients with severe acromegaly, where the skin in the axillae and back of the neck becomes dark, soft, and velvet-like with delicate folds and papillae. This phenomenon is caused by increased amounts of skin extracellular matrix, accompanied by edema. Cutaneous microcirculation is altered in patients with acromegaly. Rare manifestations include cutis verticis gyrate and psoriasis. These manifestations respond well to decreased GH level; although some changes are irreversible [14].

### 2.1. Osteoarticular Manifestations

One of the most frequent clinical manifestations of acromegaly affects the joints, in approximately 70% of individuals at the time of diagnosis. Articular alterations are the most frequent and severe cause of morbidity and disability in these patients. The pathogenesis of arthropathy in acromegaly is comprised of two mechanisms: initial endocrine elevated GH and IGF-I levels promote growth of the articular cartilage and periarticular ligaments, subsequently leading to mechanical changes. Arthralgia is one of the most common complaints of acromegalic patients. Large joint arthropathy is a common feature of the disease, occurring in approximately 70% of patients, resulting from the thickening of cartilaginous and periarticular fibrous tissue, causing joint swelling, pain, and hypomobility followed by the narrowing of joint spaces, osteophytosis, and features of osteoarthritis with chronic disease [15]. Approximately 50% of patients have axial arthropathy (disk space widening, vertebral enlargement, and osteophyte formation) mainly affecting the lumbar area. Affectation of the lumbar area can cause restricted range of movement, joint instability, and deformity of joint.

The radiological appearance of arthropathy in acromegaly was studied in small noncontrolled groups of patients with the disease treated or untreated, but active. These studies have suggested that more severe radiological changes are associated with disease duration and activity. The most prevalent manifestation was axial osteoarthritis, affecting the cervical and lumbar areas, even at young ages. The characteristic radiological changes observed were wide joint spaces and severe osteophytosis. This study documents that the risk to develop osteoarthritis is predicted by IGF-I concentrations at the time of initial diagnosis. These associations were not caused by differences in age, gender, or BMI [16]. The carpal tunnel syndrome occurs in approximately 30% to 50% of acromegalic patients and is frequently bilateral. The predominant pathology of median neuropathy in acromegaly consisted of increased edema of the median nerve in the carpal tunnel, rather than extrinsic compression due to increased volume of the carpal tunnel contents [15].

Although acromegaly is often included in lists of endocrinopathies associated with osteoporosis, due to increased bone turnover, some investigators have reported normal or increased bone mass in this disorder [17, 18]. The effects of excess GH on bone mineral density are variable in relation to the skeletal area, as a result of differing sensitivity to excess GH of the trabecular and cortical bone [19]. Decreased bone mineral density has been reported in acromegaly almost exclusively at the lumbar spine, a site rich in trabecular bone, whereas increases in bone mineral disease may be observed in the forearm, a site rich in cortical bone. The variability of data on bone mineral density in acromegaly may also be explained by the diversity of the densitometric techniques used, age and gender. Some of the inconsistencies in the literature may be accounted for as a result of the common association between acromegaly and hypogonadism. The impact of hypogonadism should therefore be considered in patients with acromegaly when evaluating bone density. GH and IGF-I have a stimulatory effect on osteoblast function, and several studies indicate a potential anabolic effect of GH, at least on the cortical bone. Altogether, these data suggest that excess GH and IGF-I induce an increase of the cortical bone density, independently of gonadal function, whereas hypogonadism seems to counteract the anabolic effect of GH on the trabecular bone [20, 21].

Chronic excess of GH and IGF-I typically affects the axial skeleton with the development of severe alterations to the spine morphology and function until features of diffuse idiopathic skeletal hyperostosis occur. An early diagnosis of acromegaly is mandatory in order to reduce the severity of spine abnormalities as they are significantly higher in patients with longer disease duration [22].

### 2.2. Cardiovascular Manifestations

Cardiovascular manifestations occur in 60% of patients. Elevated growth hormone, hypertension, and heart disease are negative determinants for life expectancy in acromegaly. Therefore controlling GH, hypertension, and heart disease are relevant in decreasing mortality rate. Cardiac involvement in acromegaly, in the absence of other contributing factors, is called acromegalic cardiomyopathy, which is initially characterized by cardiac hypertrophy, followed by diastolic dysfunction and ultimately failure of systolic function [23]. This situation is aggravated by the presence of other complications, such as diabetes or hypertension [24]. There is a reported case of death due to heart failure secondary to cardiomyopathy induced by acromegaly and aggravated by obesity [25]. The presence of arrhythmias (atrial fibrillation, supraventricular tachycardia, and ventricular arrhythmias) is also more common, especially during exercise [26]. Valvular disorders are underestimated and are related to the degree of hypertrophy [27]. Despite this unfavorable cardiovascular risk profile, it is unknown whether acromegalic patients have an increased risk of coronary artery disease.

Some authors have found an increased thickness of the intima-media layer, depending on factors such as smoking [28]. Several studies have revealed that there are atherosclerotic changes in patients with acromegaly related to the time of evolution. In a study by Colao et al. a significant increase of intima-media thickness of both common carotid arteries was observed in patients with active acromegaly, as well as in patients whose acromegaly had been cured. However, there was no increase in the prevalence of well-defined carotid plaques in either of the patient groups in comparison to controls [28]. Another study found that coronary artery disease risk in newly diagnosed acromegalic patients was low and remained stable after successful treatment [29].

In acromegalic patients, the Framingham score was calculated and screening for calcification of the coronary arteries was carried out using computed tomography. The authors found a 40% risk [30], in contrast to another study in which the cardiovascular risk calculated by the Framingham score was low [31], although both previous studies included heterogeneous groups of patients with controlled and uncontrolled disease activity. In a more recent study, cardiovascular risk factors were studied in patients with acromegaly. An increased rate of hypertension and diabetes was found, triglyceride levels were elevated, and high- and low-density lipoproteins were low. Cardiovascular risk calculation has shown that patients with acromegaly are at greater risk, particularly female patients, and that this risk decreased with normalization of IGF-I [32]. Arterial hypertension is considered to be one of the most important prognostic factors for mortality [33]. It is present in one-third of acromegalic patients, and different mechanisms are considered to be responsible, including increased plasma volume, alterations in renin-angiotensin, insulin resistance, and increased vascular resistance.

Excess GH can cause insulin resistance, as it alters the ability of insulin to suppress glucose production and stimulates its use [34]. Intolerance to carbohydrates and diabetes mellitus are frequently associated with acromegaly. The prevalence of diabetes mellitus in acromegaly is estimated to be 19 to 56%, in different series [35]. Carbohydrate intolerance occurs with a prevalence of 31% in the study by Biering et al. [36], 46% in the study of Kasayama et al. [37], and 16% in the analysis carried out by Kreze et al. [35]. The most characteristic predisposing factors for diabetes were older age and longer duration of illness. In the study of Kreze et al. [35] diabetes is influenced by a family history of diabetes and the presence of hypertension. Other factors proposed by Colao et al. [38] are the presence of concomitant hyperprolactinemia, tumor size, and a family history of diabetes. Diabetes is also associated with abnormalities in lipid metabolism, such as a tendency towards having low cholesterol levels and hypertriglyceridemia. Not all studies have identified these outcomes [39]. In most cases, when the acromegaly is cured, this leads to the disappearance of insulin resistance and therefore the disappearance of diabetes.

The possible association between intracranial aneurysms and pituitary adenoma has been frequently debated. A recent study documents an increased prevalence of intracranial aneurysms in acromegaly and the presence of intracranial aneurysms correlated with GH serum values at disease onset and showed a trend to a positive correlation with poor disease control [40].

### 2.3. Respiratory Manifestations

Acromegaly alters the structure of the respiratory apparatus and impairs respiratory function. Patients with acromegaly develop a number of respiratory alterations, as a consequence of anatomical changes affecting the craniofacial bones and soft tissues, respiratory mucosa/cartilages, lung volumes, rib cage geometry, and the activity of respiratory muscles. This range of abnormalities results in two main respiratory dysfunctions, namely, sleep apnea and impaired respiratory function. Sleep apnea, the phenomenon of recurrent cessation or reduction of airflow to the lungs during sleep, is a common cause of snoring and daytime sleepiness in acromegaly. Impaired respiratory function is less frequently investigated in acromegaly and originates from multiple alterations involving the bone and muscle structure of the chest as well as lung elasticity. Lung volumes become increased in patients with acromegaly and they may develop subclinical hypoxemia. The impact of respiratory complications is high in acromegaly, and patients with this condition may have increased respiratory mortality [41].

Patients with acromegaly develop a barrel chest due to changes in their vertebral and costal morphology. Obstruction of the upper airways is a result of macroglossia, prognathism, thick lips, and hypertrophy of the laryngeal mucosa and cartilage; it can cause sleep apnea and excessive snoring and can complicate tracheal intubation during anesthesia. Hypoventilation and hypoxemia may also arise from central respiratory depression and kyphoscoliosis. The lungs show increased distensibility with normal diffusion capacity [42].

Moreover, using polysomnography, Attal and Chanson [43] found an average rate of 69% for obstructive sleep apnea in patients with active disease in prospective or retrospective studies. Sleep apnea occurs in more than 50% of patients. In patients with acromegaly, obstructive sleep apnea predominates over central sleep apnea (which may be observed in some patients and was considered to be a consequence of an increased ventilation response to carbon dioxide) and has been linked to changes in the facial skeleton, jaw opening, and to increased laryngeal soft tissue. Another factor to consider is obesity experienced by patients with acromegaly. Medical or surgical treatment of acromegaly improves obstructive sleep apnea in a substantial number of patients, but according to several prospective studies it persists in up to 40%. This provides us with an insight into the multifactorial etiology of obstructive sleep apnea in acromegaly [44, 45].

### 2.4. Endocrinological and Other Manifestations

Hyperprolactinemia with or without galactorrhea develops in approximately 30% of patients due to of pituitary stalk compression or mixed tumor secretion of GH and PRL. Hypopituitarism, by mass compression of the normal pituitary tissue, occurs in approximately 40% patients; amenorrhea, impotence, or secondary thyroid or adrenal failure may develop. Goiter and thyroid abnormalities are common, potentially as a result of the stimulating effects IGF-I on thyrocyte growth. Hyperthyroidism rarely develops because of high levels of serum thyrotropin secreted from plurihormonal pituitary tumors. Hypercalcemia in acromegaly is reported in up to 8% of patients; it is usually secondary to coexistent hyperparathyroidism and does not resolve after treatment of excess GH. Reports indicate that hypercalciuria and nephrolithiasis may occur in 6–77% of patients with acromegaly. Proposed mechanisms of hypercalciuria include parathyroid hyperplasia, renal tubular acidosis, increased calcium absorption, and overproduction of 1, 25 (OH)_2_ D [46].

One study evaluated sleep characteristics in a small group of acromegalics; an astonishingly high number of patients were found to be affected by restless leg syndrome, a neurological disorder characterized by a compelling urge to move the limbs during the night, due to unpleasant paraesthetic sensory symptoms, which may lead to severe insomnia, consequent daytime sleepiness, and reduced quality of life. The study found that the prevalence and severity of restless leg syndrome is increased in patients with active acromegaly and impacts negatively on their physical performance, dramatically impairing quality of life [47].

### 2.5. Gastrointestinal Manifestations

The gastrointestinal manifestations associated with acromegaly are colon carcinoma, adenomatous polyps, and dolichocolon. In a recent study, colonic diverticular disease was found to be higher in patients with acromegaly when compared with controls, and diverticula were present at a significantly younger age. Diverticulosis in acromegaly was primarily associated with the duration of the active disease, which became even stronger when adjusted for excess GH and IGF-I. Dolichocolon and adenomatous polyps were increased in patients when compared with controls, both of which were associated with IGF-I concentrations at the time acromegaly was diagnosed [48].

Epidemiological studies have reported conflicting findings on cancer risk in acromegaly [49, 50]. Different studies on the risk of prostate, breast, colorectal, lung, and thyroid cancer suggest that high circulating IGF-I levels are related to an elevated risk of cancer, whereas high levels of insulin-like growth factor binding protein type 3 (IGFBP-3) levels are associated with a reduced risk. GH has a stimulatory effect on both IGF-I and IGFBP-3, and therefore there should not be an increased risk of malignancy. Excess GH causes an elevated IGF-I to IGFBP-3 ratio, which is expected to increase cancer risk. The question of whether or not elevated GH and IGF-I levels result in de novo cancer initiation remains unresolved. The cancers most frequently studied in acromegaly are colon, breast, and prostate carcinomas, although many others have been described including hematological, bronchial, gastric, esophageal, thyroid, osteosarcoma, pancreas, melanoma, ovarian, renal, adrenal, biliary, carcinoid, cervix, bladder, parotid, astrocytoma, and small intestine cancer.

Patients with acromegaly have a higher prevalence of colorectal neoplasms. The pathogenic mechanisms are still unclear and may be related to a sustained increase in serum GH-IGF-I, hyperinsulinemia, altered IGFBP-3, increased IGF-II and IGFBP-2 levels, altered local immune response, and genetic susceptibility. Elevated levels of serum IGF-I are associated with increased proliferation in the superficial crypt cells. Colao et al. investigated the relationship of GH, IGF-I, and insulin levels to colonic lesions in a cohort of consecutive newly diagnosed patients with acromegaly and found that fasting insulin levels were associated with premalignant and malignant colonic lesions. It was also found that glucose tolerance and insulin levels were strongly associated with colonic adenomas and carcinomas. Diabetes or impaired glucose tolerance was a risk factor for the development of colonic lesions [51].

Considering all the prospective colonoscopy screening studies, an increased prevalence of colorectal cancer has been found in acromegaly when compared with controls. No increase was demonstrable if acromegaly was controlled, but if the disease was active, premalignant polyps were more frequent and increased their tendency to become malignant. Once developed, the cancer was more aggressive, with higher mortality. A positive correlation between mortality from colorectal cancer and disease activity was observed. Therefore, complete colonoscopy should be offered, and at least a baseline one at diagnosis colonoscopy assessment is required in all patients with acromegaly [52]. Multiple skin tags, a positive family history or any other genetic predisposition, or advancing age are considered as predisposing features to consider colon cancer. Screening will depend on the initial findings, whether IGF-I is normal or high, and on the patient's age. Whether colonoscopy should be performed every 3 or 5 years is not defined. If acromegaly is controlled and no polyps are initially found, it is unclear whether subsequent colonoscopy is necessary at all. In summary, since there seems to be an increased prevalence of colorectal neoplasms in acromegaly, regular screening and polypectomy if required would seem advisable [53]. In principle, as there is a relationship between GH and IGF-I in serum and cancer, the most effective the treatment of acromegaly is, the less should be the mortality from colorectal cancer [54]. Further studies are necessary to evaluate the true incidence of prostate and breast cancer and hematological malignancies in acromegaly [55]. The similarity between the epithelial tissue of the gallbladder and colon raises the possibility that gallbladder polyps may occur more frequently in acromegaly. A recent study has demonstrated an increased prevalence of gallbladder polyps in patients with newly diagnosed acromegaly. Further studies are required to determine whether these patients are at increased risk of developing gallbladder carcinoma and to define the role, if any, of biliary ultrasound surveillance [56].

## 3. Diagnosis

Acromegaly is an insidious disease, which is often diagnosed late (between 4 and more than 10 years after onset). The increase in morbidity and mortality associated with acromegaly is the result of the oversecretion GH and IGF-I and the direct mass effect of the pituitary tumor. An early diagnosis of the disease is mandatory, although none of these symptoms is sufficiently sensitive, especially during early stages of the disease. Physiognomic alterations and growth of the acral parts are the first manifestations of the disease in most cases; this is the reason for a medical visit in most patients. Growth of the acral parts is uncommon in adults without acromegaly and can be objectively evaluated by an increase in shoe and/or ring size. Therefore, familiarization of physicians with the phenotype of the disease may be more effective in order to increase the diagnosis and to permit early detection, rather than strategies addressing comorbidities, which are not always present, usually appear later, and are rarely seen in the absence of the acromegaly phenotype [57]. Another possible method for early diagnosis of patients with acromegaly is to use software in order to detect the features of acromegaly, using photographs of the face. This could be a promising system for identifying the disease [58].

**Figure 1:**
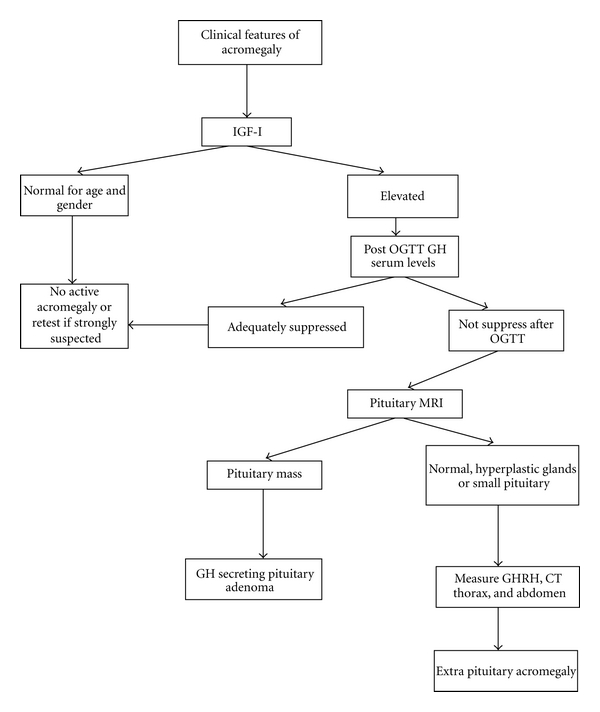
Algorithm for the diagnosis of acromegaly (modified from: Cordero and Barkan [61] and Giustina et al. [63]).

When there is clinical suspicion of the disease, biochemical confirmation is required to establish the diagnosis (Figure 1). Normal GH production from the pituitary gland is pulsatile; most GH values fall in the range of 0.1-0.2 *μ*g/L in normal subjects, with the maximum production occurring at night in harmony with sleep stages. However there are six to ten secretory bursts during the day, when GH reaches values of 5–30 *μ*g/L, which may overlap with the values seen in acromegalic patients. The diagnosis of acromegaly requires elevated levels of GH and IGF-I to be demonstrated. The best way to assess overall daily GH production is to obtain a mean GH over 24 h by frequent GH sampling, although this method is inconvenient and 24-h mean GH values have been found to overlap with those of healthy controls [59]. Initial studies assumed that a 0800 hours GH value correlated well with mean 24 h GH; however further studies revealed discrepancies. Furthermore, studies have shown that GH secretion is influenced by age and body mass index, so both diagnostic and surveillance GH levels should be interpreted in the context of age. 

GH levels are tonically elevated in acromegaly, so a random GH value of less than 0.04 *μ*g/L excludes the diagnosis [1], although an elevated random value does not imply their presence. The standard method for diagnosing acromegaly has been to measure the GH nadir during an oral glucose tolerance test (OGTT), which is undetectable in normal individuals, while acromegalics fail to suppress GH levels. It is useful to determine IGF-I levels as they correlate with the clinical features of acromegaly and with the 24-hour mean GH levels. The standard OGTT consists of the administering of 75 g of glucose with GH measurements at various time points for up to 120 minutes [60].

IGF-I has been used as a marker of disease activity in acromegaly. It is mainly synthesized in the liver, although nearly all of the tissues contribute to the circulating concentration. It is an ideal screening test as it has a long half life of 18–20 hours and the levels remain stable throughout the day. IGF-I is affected by age and gender, decreasing by approximately 14% per decade during adult life. Some disorders including malnutrition, malabsorption, anorexia nervosa, liver cirrhosis, renal failure, type 1 diabetes mellitus, normal pregnancy, and adolescence show discrepancies between GH and IGF-I [61]. The development of radioimmunoassay (RIA) provided clinicians with a biochemical tool to diagnose acromegaly. Many limitations were inherent to this methodology, which required the development of more sensitive tools, such as immunoradiometric (IRMA) or immunoluminometric (ILMA) assays for GH and IGF-I measurements. The reference ranges to describe normalcy of the somatotropic axis and the biochemical criteria of “cure” of acromegaly are areas of great debate [62].

Biochemical diagnosis is made by determining of GH after OGTT with 75 g and determining the levels of IGF-I. The current international consensus for the diagnosis of acromegaly recommends a nadir GH equal to or greater than 0.4 *μ*g/L after an OGTT, in conjunction with clinical suspicion and high IGF-I levels [63]. The diagnosis is sometimes complicated by the physiological variations of GH and the lack of uniformity of methods for GH determination. The new monoclonal antibody-based immunoassays are more sensitive, although they suffer from significant problems in terms of reproducibility [64]. GH cannot be suppressed in the presence of liver failure, kidney failure, poorly controlled diabetes, malnutrition, anorexia, pregnancy, estrogen therapy, or in late adolescence [1]. In contrast, a recent study [65], found that 7 adult patients with newly diagnosed untreated acromegaly out of a total group of 40 had a GH nadir after an OGTT of less than 0.4 *μ*g/L. These data highlight the limited diagnostic value of OGTT in patients with biochemically active acromegaly but only mildly increased GH output and the limitations for the applicability of consensus guidelines on diagnosis in clinical practice [66].

In different situations IGF-I serves as a biomarker of the activity of acromegaly. IGF-I levels are relatively stable and correlate with clinical acromegaly and elevated GH levels. In order to accurately assess IGF-I levels, age-matched controls are required, as the levels of IGF-I decreased 14% per decade. In order to monitor disease activity, levels of GH and IGF-I are complementary. It has been suggested that in the presence of discordant serum IGF-I and GH levels, IGF-I is more predictive than GH in terms of insulin sensitivity and clinical symptom score [67]. The measurement of the acid-labile subunit, other binding proteins (such as IGF-binding protein-3), or ghrelin offers no advantage over IGF-I measurement in the diagnosis and management of acromegaly [63].

As previously mentioned, pituitary GH-secreting tumors may cosecrete other hormones. Hyperprolactinemia may be due to cosecretion or pituitary stalk compression. Mass effect of the tumor may affect the secretion of the pituitary gland hormones, resulting in hypopituitarism. Therefore, other pituitary hormones and their target hormones should be evaluated.

Magnetic resonance imaging (MRI) with contrast administration is the best imaging technique to pinpoint the pituitary source of excess GH. This technique makes it possible to visualize and locate, in relation to surrounding structures, adenomas larger than 2 mm in diameter. At the time of diagnosis, over 75% show a macroadenoma (>10 mm in diameter), which grows into the cavernous sinus or suprasellar region. In rare cases, in patients with acromegaly and unremarkable pituitary MR imaging and no evidence of ectopic GH or GHRH production (CT or MRI thoracic and abdominal negative), a transsphenoidal pituitary exploration is a reasonable approach and may result in clinical improvement and biochemical cure in the hands of experienced surgeon [68].

As previously discussed, acromegaly is rarely caused by ectopic tumors that produce GHRH (only 0.5% of acromegalic cases) or GH [8]. The suspicion for ectopic sources should arise when there is a biochemical diagnosis of acromegaly, with radiographic absence of a pituitary tumor or presence of diffuse pituitary enlargement or when the postsurgical pathology report indicates somatotroph hyperplasia rather than adenoma. The measurement of plasma GHRH concentrations can be very helpful. Total body scintigraphy with radio-labeled somatostatin should be performed in order to localize the tumor. A definitive diagnosis of ectopic GHRH production can be made either by showing an arteriovenous gradient of GHRH across the tumor bed or by normalization of GHRH, IGF-I, and GH levels after removal of the tumor [69]. Figure 1 illustrates the algorithm for the diagnosis of acromegaly.

It has been suggested that the mortality rate in patients with acromegaly is correlated with the degree of control of GH. The mortality rate from cancer can be stratified according to GH levels aftercare and may be similar to that of the nonacromegalic population. The standardized mortality rate for acromegaly is 1.72. Mortality is related to GH levels of over 2–2.5 *μ*g/L and less clearly with elevated IGF-I [70]. Treatment with radiotherapy may be associated with increased mortality [71]. A meta-analysis of mortality ratios in patients with acromegaly found all-cause mortality increased in comparison to the general population. Although the mortality risk has decreased due to modern treatment strategies, including transsphenoidal surgery, there is still a 32% increased all-cause mortality risk in acromegaly. Further investigation is required in order to determine whether newer treatment strategies in acromegaly are accompanied by a further improvement in survival [72].

Clinical and biochemical control is aimed at correcting tumor compression, controlling biochemical markers to normal levels, and reducing morbidity and mortality to the expected rate for the normal population. According to the 2010 consensus criteria, biochemical control of acromegaly is achieved when circulating IGF-I is reduced to an age- and sex-adjusted normal range and GH during OGTT is <0.4 *μ*g/L or random GH is <1 *μ*g/L [63]. The prognosis of acromegaly has improved in recent years, and adequate hormonal disease control is achieved in most cases, allowing life expectancy similar to that of the general population [63].

## 4. Conclusion

Acromegaly is an uncommon disease, which in most cases is due to a pituitary tumor. It has a wide variety of clinical manifestations, including acral and soft tissue overgrowth, joint pain, diabetes mellitus, hypertension, and heart and respiratory failure. Acromegaly is usually diagnosed by increased IGF-I and GH after an OGTT. Its treatment reduces complications and mortality.

## Figures and Tables

**Table 1:** Clinical manifestations of acromegaly.

Mass effects of the tumor
Headache
Visual field defects
Hyperprolactinemia
Pituitary stalk section
Hypopituitarism
Hypothyroidism, hypogonadism, hypocortisolism
Systemic effects of GH/IGF-I excess
Visceromegaly
Soft tissue and skin changes
Thickening of acral parts
Increased skin thickness and soft tissue hypertrophy
Hyperhidrosis/Oily texture
Skin tags and acanthosis nigricans
Cardiovascular features
Hypertrophy (biventricular or asymmetric septal)
Congestive Heart Failure (systolic and/or diastolic)
Coronary disease
Arrhythmias
Hipertensión
Cardiomyopathy
Metabolic features
Impaired glucose tolerance
Diabetes mellitus
Insulin resistance
Respiratory manifestations
Macroglossia
Jaw malocclusion
Upper airway obstruction
Sleep disturbances
Sleep apnea (central and obstructive)
Ventilatory dysfunction
Bone and joint manifestations
Increased articular cartilage thickness
Arthralgias and arthritis
Carpal tunnel síndrome
Osteopenia
Other endocrine consequences
Goiter
Hypercalciuria
Galactorrhea
Decrease libido, impotents
Menstrual abnormalities

Modified from Cordero and Barkan [61].
